# Database of segmentations and surface models of bones of the entire lower body created from cadaver CT scans

**DOI:** 10.1038/s41597-023-02669-z

**Published:** 2023-11-03

**Authors:** Maximilian C. M. Fischer

**Affiliations:** RWTH Aachen University, 52062 Aachen, Germany

**Keywords:** Bone, Image processing

## Abstract

The range of applications of digital surface models of the bones in science and industry is wide. Three-dimensional reconstructions of bones are used in biomechanics, biomedical engineering, medical image processing, orthopedics, traumatology, radiology, patient education, anatomy, anthropometry, forensic anthropology, ergonomics, usability and human factors engineering, or accident and injury analysis and prevention. No open access database or repository of skeletal surface models of the full lower extremities exists. Therefore, the objective of this publication was to provide access to consistent complete bone models of the pelvis and lower limbs of multiple subjects, including biometric data. Segmentations and surface models of the bones of the lower extremities of more than twenty subjects were created from open access postmortem whole-body computed tomography scans. The database provides a broad range of applications by giving access to the data of the complete process chain, from the raw medical imaging data through the segmentations to the surface models.

## Background & Summary

The field of application of digital bone models is broad. Three-dimensional (3D) reconstructions of bones are used in biomechanics, biomedical engineering and medical image processing for musculoskeletal modelling^1,2^, finite element analyses^3^, statistical shape modelling^4–6^ or 3D reconstruction from sparse imaging data, such as radiographs^7,8^ or EOS images^9^. 3D reconstructions of the bones are used in orthopedics, traumatology or radiology for the development of implants^10–14^, surgical instruments^15,16^ or procedures, for diagnosis and decision-making^17,18^, preoperative planning^19,20^ and navigational guidance during computer assisted surgery^8,21^, the evaluation of outcome^22^, surgery simulation^23^, surgical education and training^24^, especially in the context of personalized, patient-specific, customized or individualized medicine. The surgical guidance based on bone models can be virtual, augmented^25^ or mixed reality^26^, or 3D printed^27,28^. Further fields of application are anatomy and patient education^29,30^, morphometrics^31^ and anthropometry^32,33^, forensic anthropology^34,35^, ergonomics, usability and human factors engineering^36^, accident and injury analysis and prevention^37^.

However, to the best of the author’s knowledge, no open access database or repository of skeletal surface models of the full lower extremities exists. Therefore, the objective of this study was to provide access to consistent complete bone models of the pelvis and lower limbs of multiple subjects. The database is supposed to enable other researches to quickly develop, test and verify new methods, approaches, algorithms or proofs of concept without the time-consuming and labor-intensive work of data collection and curation, segmentation and reconstruction. The database is expected to help the scientific community to facilitate research and improve the reproducibility and comparability of studies by giving access to the raw medical imaging data, including the metadata of the subjects and the segmentations and surface models of the bones. Hence, different researchers and research groups can resort to the same datasets for the validation of methods and comparison of results. Different deep learning models for artificial intelligence-based bone reconstruction, for instance, could be benchmarked by applying them to the raw computed tomography (CT) data and comparing the automatic with the manual segmentations of the database. The database can also be used as additional training data for existing deep learning models^38,39^.

## Methods

### Source of the raw CT data

The segmentations and models of the bones of the lower extremities were created from anonymized postmortem CT scans of the whole body originally published by Kistler *et al*. in the Swiss Institute for Computer Assisted Surgery Medical Image Repository (smir.ch) as open access Virtual Skeleton Database (VSD)^40^. The CT datasets were provided by the forensic institutes of the universities of Bern and Zürich and shared under the Creative Commons Attribution-NonCommercial-ShareAlike (CC BY-NC-SA) license after ethical approval of the Cantonal Ethics Committee Bern^41^. Further information about the datasets can be found in the literature cited^40,41^. Due to ongoing difficulties in accessing the SMIR website, the author decided to reupload the original datasets without any changes to the open access hosting service Zenodo: 10.5281/zenodo.8270364^42^.

#### CAUTION

The VSD contains a few inconsistencies, such as duplicate CT datasets. The author of this publication is not connected to the SMIR or VSD and, therefore, not responsible for errors in the VSD. However, errors that the author recognized during the work with the VSD were logged and are reported in the reupload of the VSD^42^.

### Subject selection

Twenty subjects (ten male and ten female) were selected from the VSD for the creation of the bone models with the objective of covering a wide age range.

The inclusion criteria were:

- Availability of age, body weight and body height.
- Integrity and completeness of the lower body’s skeletal anatomy.

The exclusion criteria were:

- Difference between the gender specified in the metadata and the biological sex visible in the CT data.
- Presence of artificial joints or bone fractures.

The average age, weight and height of the twenty subjects were 52 ± 21 years, 70 ± 13 kg and 1.7 ± 0.1 m, respectively. An overview of the subjects is presented in Table 1. Some subjects were processed before the inclusion and exclusion criteria were defined. Ten of the subjects did not meet the criteria. These ten additional subjects are also published as part of the database since they still might be useful for some applications, but they are tagged by a comment in the database so they can be easily identified by the user (see Table 1).

**Table 1:** Twenty complete subjects of the database and ten additional incomplete or inconsistent subjects.

ID	Age [years]	Sex	Weight [kg]	Height [m]	Comment
**Twenty complete subjects**					
002	78	F	75	1.62	
006	51	F	90	1.77	
010	45	F	54	1.65	
014	30	F	65	1.65	
015	81	M	78	1.75	
016	95	F	60	1.52	
017	19	F	59	1.7	
019	56	M	68	1.7	
023	74	M	86	1.82	
z001	76	M	87	1.8	
z004	65	M	82.3	1.77	
z009	25	M	74	1.75	
z019	58	M	71.3	1.81	
z023	47	F	61	1.66	
z027	37	F	51.5	1.69	
z035	30	F	50.45	1.68	Duplicate of VSD z030.
z042	61	F	53.4	1.69	
z046	38	M	72	1.8	
z056	26	M	81.8	1.87	
z062	43	M	76.95	1.77	
**Ten additional incomplete or inconsistent subjects**					
z013	41	F	56.3	1.65	Duplicate of VSD z024 with conflicting metadata. Intraosseous access in the left tibia.
z036	62	M			Duplicate of VSD z029. Missing body weight and height.
z049	34	M	87	1.79	Difference between the gender specified in the metadata and biological sex visible in the CT data.
z050	84	M	73.4	1.67	Duplicate of VSD z011. Hinged TKR of the right knee joint.
z055	73	M	73	1.73	Duplicate of VSD z026 with conflicting weight information in the metadata.
z057	75	M			Missing body weight and height.
z061	39	F	37.4	1.8	Right phalanges are cut off.
z063	72	F	80.2	1.72	Spinal fusion of L4-L5-S1. THR of the left and right hip joint. TKR of the right knee joint.
z064	69	M			Missing body weight and height.
z066	48	M			Metacarpals are cut off and phalanges are missing. Metal artifacts. Missing body weight and height.

“Sex” refers to the biological sex visible in the CT data. THR = total hip replacement, TKR = total knee replacement.

### Reconstruction of the osseous anatomy

**Fig. 1:**
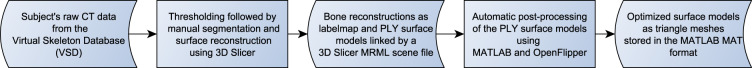
Workflow of the creation of the lower body’s bony anatomy surface models.

The bone surfaces were semi-automatically reconstructed by thresholding (Fig. 1). Two hundred Hounsfield units^43^ were chosen as the lower threshold and the maximum Hounsfield unit value present in the volume data was selected as the upper threshold. Subsequently, a manual post-processing using the software 3D Slicer (slicer.org) with default smoothing settings was performed^44^. The bones were manually segmented at the joints if necessary. All joints were segmented. However, some segments contain multiple components as follows:

- Sacrum including the coccyx (if not fused with the sacrum)
- Hip bone (also called pelvic, innominate or coxal bone)
- Femur
- Patella
- Tibia
- Fibula
- Talus
- Calcaneus
- Tarsals, including the cuboid, navicular and three cuneiforms
- Metatarsals
- Phalanges

Separate segments were created for the left and right leg. Some segments contain small sesamoid bones if present. This applies to the metatarsals for all subjects but, in some cases, also to other bones, such as the femurs.

After the segmentation, the bones were reconstructed by manually closing holes present in the outer surface. No gap closing, hole filling or wrapping algorithms were used. The reconstructed surface models were exported as mesh files in the Polygon File Format (PLY) and imported into MATLAB using a conservative decimation and remeshing procedure (Fig. 1). The Hausdorff distance between input and output mesh was limited to 0.05 mm for the decimator. The adaptive remesher permitted a maximum deviation of 0.05 mm from the input mesh with a minimum and maximum edge length of 0.5 and 100 mm, respectively. The decimator and remesher are plugins of the software OpenFlipper (openflipper.org)^45^.

#### CAUTION

Each reconstruction of anatomical structures from medical images is subject to cumulative spatial errors arising from each step of the process chain. While the section “Technical Validation” should give an impression of the error that can be expected from the workflow described, users of the database should take into account the risk of larger reconstruction errors depending on the application intended.

**Fig. 2:**
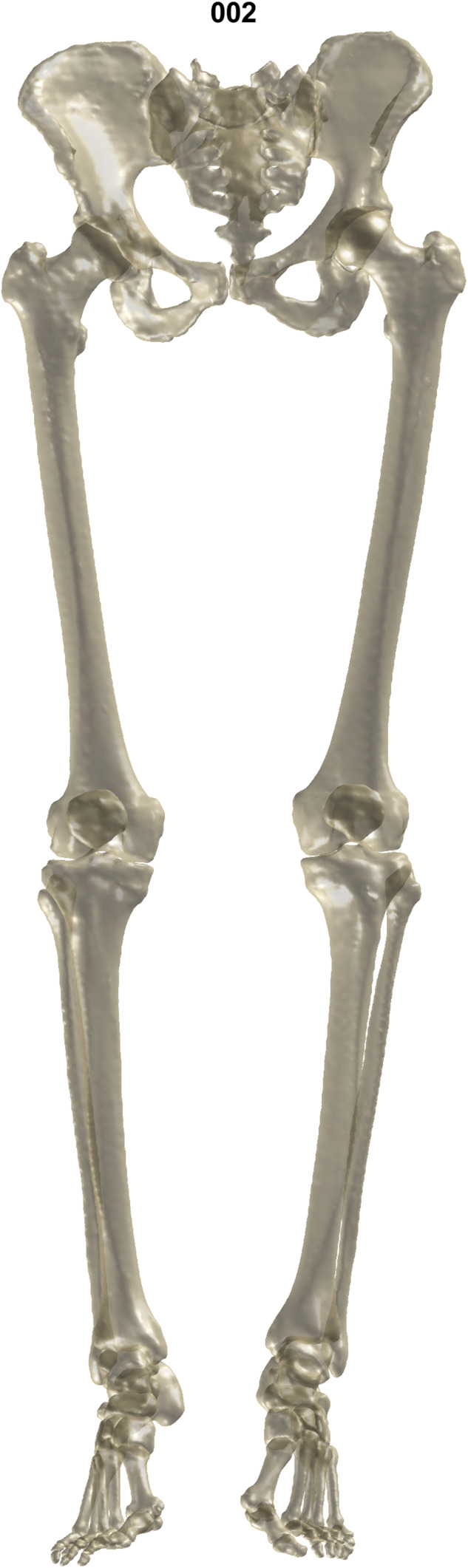
Surface models of the lower body’s osseous anatomy of subject 002.

The bone models of each subject can be visualized by running the MATLAB or Python examples. One subject is presented in Fig. 2. The 3D reconstructions were created by the author as a private side project between 2017 and 2022. Parts of the database containing fewer subjects and only the pelvis and femurs were published previously as part of other studies of the author^46,47^. This research received no specific grant from any funding agency in the public, commercial or not-for-profit sectors.

### Analysis of the surface models stored as MAT files

The database was searched for duplicate subjects using a two-stage registration process. Each pelvis was transformed into an automatically detected pelvic coordinate system based on the anterior pelvic plane using the iterative tangential plane method^46^. Subsequently, the sacrum of each subject was registered to the sacra of all other subjects using a rigid iterative closest points algorithm. Lower outliers of the root mean square error between the two registered sacra were examined. One duplicate subject was identified, excluded from the database and replaced by another subject.

Each bone model of all subjects was visually reviewed for internal cavities connected to the outer surface or connections between the inner and outer surface, and corrections were performed if necessary. The mesh topology was checked for the following errors using MATLAB:

- Duplicate, non-manifold and unreferenced vertices.
- Boundary, non-manifold and conflictingly oriented edges.
- Duplicate and degenerated faces.
- Self-intersections and intersections with adjacent bones.

The errors were corrected if present.

The volume enclosed by the outer surface of the bone models was calculated and is presented in Table 2. The values were compared with those from literature. However, caution must be applied since different definitions and measurement methods of the bone volume exist. Studies reporting the trabecular or cortical volume of the bones were not considered. The values of the bone volume correspond to those observed in previous studies^48–51^.

**Table 2:** Volume enclosed by the outer surface of the bone models of the twenty complete subjects of Table 1.

Bone name	Volume [cm^3^]											
	All 20 subjects				10 male subjects				10 female subjects			
	Min.	Mean ± SD	Median (IQR)	Max.	Min.	Mean ± SD	Median (IQR)	Max.	Min.	Mean ± SD	Median (IQR)	Max.
Sacrum	159	203 ± 27	198 (35)	258	159	209 ± 27	216 (42)	242	159	198 ± 27	193 (29)	258
Hip_R	256	328 ± 56	313 (90)	454	299	365 ± 52	383 (83)	454	256	290 ± 28	293 (39)	340
Hip_L	239	328 ± 55	313 (91)	448	299	364 ± 50	382 (81)	448	239	292 ± 31	291 (50)	336
Femur_R	382	533 ± 113	545 (214)	772	550	626 ± 66	633 (94)	772	382	440 ± 57	418 (59)	540
Femur_L	369	535 ± 114	547 (210)	762	556	632 ± 57	634 (46)	762	369	438 ± 55	423 (64)	538
Patella_R	11	19 ± 5	19 (7)	31	19	23 ± 4	22 (6)	31	11	15 ± 2	15 (3)	18
Patella_L	12	19 ± 5	19 (7)	32	19	23 ± 4	22 (4)	32	12	16 ± 3	16 (4)	20
Tibia_R	235	334 ± 74	354 (135)	484	355	396 ± 39	395 (47)	484	235	271 ± 37	261 (41)	352
Tibia_L	237	337 ± 73	354 (139)	479	353	399 ± 36	404 (32)	479	237	276 ± 38	264 (39)	358
Fibula_R	40	62 ± 16	63 (21)	104	62	73 ± 13	70 (13)	104	40	50 ± 8	48 (12)	66
Fibula_L	38	61 ± 15	60 (20)	104	59	71 ± 13	66 (10)	104	38	51 ± 9	48 (12)	68
Talus_R	26	37 ± 9	36 (17)	54	35	44 ± 6	46 (9)	54	26	30 ± 5	29 (5)	43
Talus_L	25	38 ± 9	35 (16)	54	35	45 ± 6	45 (5)	54	25	31 ± 6	30 (4)	47
Calcaneus_R	51	70 ± 14	69 (24)	102	62	80 ± 11	81 (7)	102	51	59 ± 8	57 (11)	78
Calcaneus_L	49	70 ± 14	69 (22)	99	62	80 ± 10	82 (8)	99	49	60 ± 9	59 (13)	77
Tarsals_R	31	43 ± 9	44 (16)	60	42	51 ± 6	49 (9)	60	31	36 ± 6	34 (7)	50
Tarsals_L	29	44 ± 10	43 (17)	62	40	51 ± 6	50 (8)	62	29	36 ± 7	35 (6)	52
Metatarsals_R	37	49 ± 10	48 (17)	67	44	57 ± 6	56 (5)	67	37	41 ± 4	39 (5)	51
Metatarsals_L	35	49 ± 9	48 (17)	66	44	57 ± 5	56 (3)	66	35	41 ± 4	39 (4)	51
Phalanges_R	12	16 ± 3	15 (5)	23	14	18 ± 3	18 (3)	23	12	13 ± 1	13 (1)	16
Phalanges_L	12	16 ± 3	15 (5)	25	13	18 ± 3	18 (2)	25	12	13 ± 1	13 (2)	16

R = right, L = left.

## Data Records

As mentioned above, a mirror of the complete VSD as hosted originally by Kistler *et al*. at smir.ch is available at Zenodo: 10.5281/zenodo.8270364^42^.

The CT volume data, segmentations, reconstructions and raw PLY mesh files of the subjects of Table 1 are accessible via Zenodo: 10.5281/zenodo.8302448^52^. The files of each subject are linked by a project file, called MRML scene file, that can be opened with the open-source medical imaging software 3D Slicer (slicer.org).

The post-processed mesh files of the subjects of Table 1 are stored as MATLAB MAT files, released as Git repository at https://github.com/MCM-Fischer/VSDFullBodyBoneModels and versioned via Zenodo: 10.5281/zenodo.8316730^53^. The use of the MAT files is explained by examples for MATLAB and Python in the Git repository.

## Technical Validation

The VSD also contains CT data of the European Spine Phantom that was introduced by Kalender *et al*. in 1995^54^. The CT phantom data was used to evaluate the reconstruction process described above. After the creation of the surface model of the phantom, landmarks and areas were manually selected on the surface model of the phantom. Planes or cylinders were fitted to the areas selected to calculate the geometric parameters of the phantom. The errors between the reconstructed and the reference values of the geometric parameters reported in the publication by Kalender *et al*. are presented in Table 3. The mean error was 0.2 ± 0.4 mm and the mean absolute error was 0.4 ± 0.2 mm. This agrees well with accuracies reported in literature for 3D bone reconstruction using CT. Lalone *et al*. reported a mean error of 0.4 ± 0.3 mm for the cortical bone of the upper extremities^55^, Wang *et al*. reported a mean error of 0.5 ± 0.2 mm for machined bone specimens from the femur and tibia^56^ and van den Broeck *et al*. reported a mean absolute error of 0.5 ± 0.2 mm for the tibia^57^.

**Table 3:** Differences between the reconstructed values and the reference values of the geometric parameters of the European Spine Phantom^54^.

All values in [mm]	Reconstructed values			Reference values^54^			Error		
	Low	Medium	High	Low	Medium	High	Low	Medium	High
Body diameter	36.64	36.66	36.63	36.0	36.0	36.0	0.64	0.66	0.63
Arch diameter*		28.47			28.0			0.47	
Body height	25.25	25.40	25.39	25.0	25.0	25.0	0.25	0.40	0.39
Arch thickness	5.33	5.97	7.24	5.2	6.0	7.0	0.13	−0.03	0.24
Spinous process thickness	5.79	8.12	10.33	6.0	8.0	10.0	−0.21	0.12	0.33
Spinous process length	11.36	14.12	20.49	11.7	14.6	21.0	−0.34	−0.48	−0.51

*Only one cylindrical fit was performed for the arch diameter, including the low, medium and high vertebra.

## Data Availability

The code used to create and analyze the datasets is openly accessible via https://github.com/MCM-Fischer/VSDFullBodyBoneModels and versioned at Zenondo: 10.5281/zenodo.8316730^53^.
